# Ecology and geography of hemorrhagic fever with renal syndrome in Changsha, China

**DOI:** 10.1186/1471-2334-13-305

**Published:** 2013-07-03

**Authors:** Hong Xiao, Xiaoling Lin, Lidong Gao, Cunrui Huang, Huaiyu Tian, Na Li, Jianxin Qin, Peijuan Zhu, Biyun Chen, Xixing Zhang, Jian Zhao

**Affiliations:** 1 College of Resources and Environment Science, Hunan Normal University, Changsha, 410081, China; 2 Hunan Provincial Center for Disease Control and Prevention, Changsha, 410002, China; 3 Centre for Environment and Population Health, School of Environment, Griffith University, Brisbane, Queensland, 4111, Australia; 4 West China School of Public Health, Sichuan University, Chengdu, 610041, China; 5 Changsha Municipal Center for Disease Control and Prevention, Changsha, 410001, China; 6 Peking University Health Science Center, Beijing, 100191, China

## Abstract

**Background:**

Hemorrhagic fever with renal syndrome (HFRS) is an important public health problem in mainland China. HFRS is particularly endemic in Changsha, the capital city of Hunan Province, with one of the highest incidences in China. The occurrence of HFRS is influenced by environmental factors. However, few studies have examined the relationship between environmental variation (such as land use changes and climate variations), rodents and HFRS occurrence. The purpose of this study is to predict the distribution of HFRS and identify the risk factors and relationship between HFRS occurrence and rodent hosts, combining ecological modeling with the Markov chain Monte Carlo approach.

**Methods:**

Ecological niche models (ENMs) were used to evaluate potential geographic distributions of rodent species by reconstructing details of their ecological niches in ecological dimensions, and projecting the results onto geography. The Genetic Algorithm for Rule-set Production was used to produce ENMs. Data were collected on HFRS cases in Changsha from 2005 to 2009, as well as national land survey data, surveillance data of rodents, meteorological data and normalized difference vegetation index (NDVI).

**Results:**

The highest occurrence of HFRS was in districts with strong temperature seasonality, where elevation is below 200 m, mean annual temperature is around 17.5°C, and annual precipitation is below 1600 mm. Cultivated and urban lands in particular are associated with HFRS occurrence. Monthly NDVI values of areas predicted present is lower than areas predicted absent, with high seasonal variation. The number of HFRS cases was correlated with rodent density, and the incidence of HFRS cases in urban and forest areas was mainly associated with the density of *Rattus norvegicus* and *Apodemus agrarius*, respectively.

**Conclusions:**

Heterogeneity between different areas shows that HFRS occurrence is affected by the intensity of human activity, climate conditions, and landscape elements. Rodent density and species composition have significant impacts on the number of HFRS cases and their distribution.

## Background

Hemorrhagic fever with renal syndrome (HFRS) is a group of rodent-borne diseases caused by hantaviruses (HV). It is an important public health problem in China, with 30,000 to 60,000 cases reported annually, accounting for 90% of human cases reported globally over the last 10 years [1]. In China, HFRS is caused mainly by two types of hantavirus, Hantaan virus (HTNV) and Seoul virus (SEOV), each of which has co-evolved with a distinct rodent host [2]. HTNV is carried by *Apodemus agrarius*, and SEOV by *Rattus norvegicus*. Humans usually become infected with hantaviruses through contact with or inhalation of aerosols and secretions from infected rodent hosts.

Hunan Province has become one of the most severely endemic areas in China since the first case was discovered in 1963. It is a traditional and mixed epidemic area, with SEOV the main hantavirus type and *Mus musculus*, *R. norvegicus* and *A. agrarius* the dominant rodent species. The incidence rate of HFRS peaked at 101.68/100,000 in 1994, and it has become a great threat to the health of the local population. Previous studies have found that precipitation, temperature, humidity, normalized difference vegetation index (NDVI), and land use types are important risk factors for HFRS incidence [3-6].

In recent years, ecological niche models (ENMs) have been developed and used to examine associations between spatial risk factors and ecological niches in disease transmission [7]. Species distributions are constrained by a series of evolutionary adaptations that are generally conceptualized as the ecological niche. Disease transmission involves interacting species (pathogens, vectors and hosts), and the conjunction of their individual ecological niches determines patterns of transmission. ENMs can be used to analyze geographic and ecological information, and then quantify potential risks based on vector, host and pathogen data [8,9]. To characterize geographic patterns of disease transmission, traditional methods usually summarize overall patterns and trends in the form of a smoothed surface, at some level of generalization or averaging. This may involve loss of resolution and may not take into account the fine-scale ecological variation that underlies transmission patterns. However, ENMs permit fine-scale characterization of geographic patterns without loss of resolution [7,10].

The characteristics of HFRS vary with locality, owing to the vast and complex topography, intricate ecological environments, and varied climates of China. It is important to identify the risk factors of HFRS and predict high risk areas, thereby facilitating application of prevention strategies to avoid or decrease loss of life. Since rodent population density and species composition have significant impacts on HFRS occurrence, it is of great importance to analyze the relationship between rodent host and HFRS incidence. In this study, we investigated the effects of ecological and geographic factors on HFRS occurrence using ENMs, and explored the relationship between HFRS cases and rodent hosts by the Markov chain Monte Carlo (MCMC) method, using data from Changsha, China.

## Methods

### Study area

**Figure 1:**
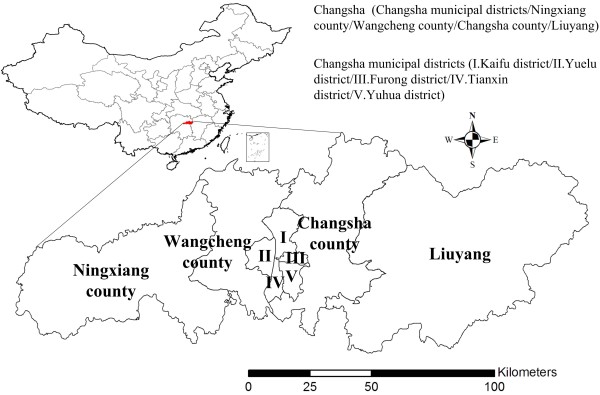
Location of study area, showing city of Changsha in mainland China.

Changsha is the capital city of Hunan Province in central China, between 27°51'– 28°40'N and 111°53'– 114°5'E (Figure 1). Changsha is about 233 km long and 90 km wide, with a total land area of 118,000 km^2^ and population of 6 million. Changsha is composed of the alluvial plain of the Xiangjiang River, which is a part of the Central China Plain. The Xiangjiang and its branches flow throughout the city, and cultivated land is widely distributed along the rivers. Subtropical double-harvest rice is the main crop, and most farmers reside less than 50 m from their farmlands. Traditional farming methods provide an opportunity for wild rodent propagation, offering suitable living conditions and sufficient food resources to increase transmission of HFRS between rodents and from rodents to humans. Furthermore, HFRS rodent hosts are widely distributed in Changsha, including *A. agrarius*, *R. norvegicus*, *M. musculus*, *Rattus flavipectus* and *Rattus rattoides*. The infection rate of hantaviruses in some rodent species is relatively high; e.g., the rates of *R. norvegicus* and *A. agrarius* in 2005 were 3.17% and 2.80%, respectively. Meanwhile, a dense human population resulting from rapid urbanization in the Changsha-Zhuzhou-Xiangtan metropolis has increased interaction between people infected with hantavirus and those susceptible to infection. These factors provide an opportunity for HFRS to persist and spread in Changsha. Since 2005, Ningxiang County has been established as a national surveillance site to monitor the relationship between humans and HFRS host animals.

### Ethical review

The study protocol was reviewed by the institutional review board of the Hunan CDC and ethics approval was not required.

### Data collection and management

**Figure 2:**
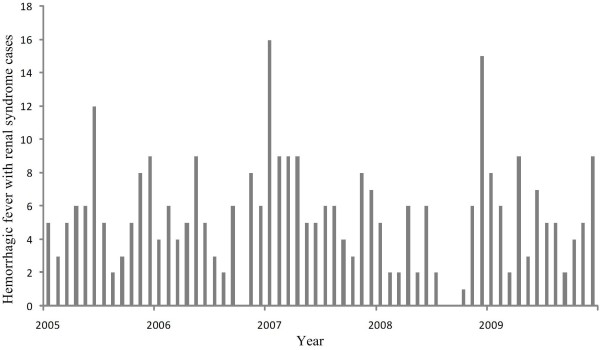
Monthly HFRS incidence in Changsha, from 2005 through 2009.

Data on reported HFRS cases in Changsha from 2005 to 2009 (Figure 2) were obtained from the Hunan Notifiable Disease Surveillance System (HNDSS) [11]. There were a total of 327 case reports, which included the sex, age and residential address of each patient, plus onset date of symptoms for each case. The HNDSS HFRS data do not differentiate HTNV from SEOV infections. All cases were geocoded by residential address at town or village level using Google Earth. First, the city of Changsha was entered, and then the name of the county or district. Finally, a more specific address, the name of town or village, was added. To ensure accuracy of the geocoding results, Google Maps was also used in the same way.

Surveillance on hantavirus infections among rodent hosts was carried out in Ningxiang County, according to the protocol established by Chinese Centers for Disease Control and Prevention. Representative villages were selected for rodent surveillance, according to the distribution of HFRS epidemics and landscape elements. Indoor and outdoor rodent traps with peanuts were set at night, and recovered in the morning. The traps were placed about 1 km away from villages in locations where rodents were most likely found, such as the edges of rivers and roads, on ridges and in yards. Traps were set in spring (March to April) and autumn (September to October). More than 100 traps per patch were placed indoors at approximately 12–15-meter intervals for three consecutive nights, and more than 200 traps per patch were placed outdoors (every 5 meters in each row, with 50 meters between rows). A total of 22,060 traps were set, and 728 rodents were captured from 2005 to 2009. Relative population density of rodents was used as an indicator of abundance. This is calculated as the number of rodents captured, divided by the number of traps.

Data on eco-geographical variables (possibly contributing to hantavirus transmission among rodent hosts) were collected and processed in ArcGIS 9.3 (ESRI Inc., Redlands, CA, USA) (Table 1). Data for each variable were converted to the same geographic projection (Gauss-Kruger-Xian 1980) and clipped to the study area. Data on elevation, precipitation, temperature, precipitation seasonality and temperature seasonality were obtained from WorldClim (http://www.worldclim.org/). Slope and aspect values were calculated from elevation in ArcGIS 9.3. Land use data were obtained from the Second National Land Survey (http://www.mlr.gov.cn/zt/dierciquanguotudidiaocha/index.htm). The compound topographic index (CTI, also called topographic wetness index), a function of both slope and upstream contributing area per unit width orthogonal to the flow direction, was obtained from the United States Geological Survey (http://eros.usgs.gov). The Human Footprint Index was obtained from the Center for International Earth Science Information (http://www.ciesin.columbia.edu/). Population in 2005 was obtained from the Changsha Statistical Yearbook, and population density calculated at the district and county level. The NDVI value from 2005 to 2009 was obtained from the National Aeronautics and Space Administration (http://ladsweb.nascom.nasa.gov/data/), and then average monthly values were calculated. All environmental variables were resampled to raster data with a spatial resolution of 0.00833° (nearly 1 km).

**Table 1:** Ecogeographical variables for modeling

**Variables**	**Data source**	**Type**
Temperature	WorldClim (http://www.worldclim.org/)	Continuous
Temperature seasonality	WorldClim (http://www.worldclim.org/)	Continuous
Precipitation	WorldClim (http://www.worldclim.org/)	Continuous
Precipitation seasonality	WorldClim (http://www.worldclim.org/)	Continuous
Elevation	WorldClim (http://www.worldclim.org/)	Continuous
Slope	Derived from elevation	Continuous
Aspect	Derived from elevation	Categorical
Land use	The Second National Land Survey Data	Categorical
CTI	United States Geological Survey (http://eros.usgs.gov)	Continuous
Human Footprint index	International Earth Science Information Center(http://www.ciesin.columbia.edu/)	Continuous
Population	Changsha Statistical Yearbook	Continuous
MHT	World Wildlife Fund(http://conserveonline.org)	Categorical
NDVI	National Aeronautics and Space Administration (http://ladsweb.nascom.nasa.gov/data/)	Continuous

### Ecological niche models

ENMs were developed to reflect the internal relationship between environmental variation and the distribution of HFRS. ENMs simulate and predict disease risks by exploring the relationship between the disease pattern and landscape elements such as land use type, temperature, precipitation, and elevation [9]. We used the Genetic Algorithm for Rule-set Production (GARP) for ENMs development. Occurrence points were divided randomly into three parts: extrinsic testing data (50%), used for model evaluation; training data (25%), used for model development; intrinsic testing data (25%), used for intrinsic evaluation and tuning of model rules. Distributional data were converted to raster layers. Then, 1,250 points randomly sampled from the training and intrinsic testing data and 1,250 points randomly sampled from the study region were used to calculate predictive accuracy (Table 2). GARP works iteratively for rule selection: a method is chosen from a set of possibilities (logistic regression, bioclimatic rules, atomic rules and range rules), and it is then applied to the training dataset to develop or evolve a rule. Rules evolve in ways that mimic DNA evolution (e.g., point mutations, deletions and crossing over). At each iteration, the change of predictive accuracy (Table 2) from one iteration to the next is used to evaluate a particular rule [12,13]. The ecological niche modeling here was done using a desktop version of GARP, which is publicly available (http://www.nhm.ku.edu/desktopgarp).

**Table 2:** Test methods of ecological niche models

**Testing**	**Formula**
Overall Accuracy	(a + d) / (a + b + c + d)
False Negatives	c / (a + c)
False Positives	b / (b + d)

a = areas of actual presence predicted present; b = areas of actual absence predicted present; c = areas of actual presence predicted absent; d = areas of actual absence predicted absent.

To build different subset models for the entire occurrence area, an algorithm threshold of 0.01 was selected, with 1,000 iterations as an upper limit for each replicate. Because of the stochastic nature of GARP in producing different outputs at different replicates, best practice approaches were required [14]. Ten best subsets were selected, with a threshold level of 0% extrinsic hard omission and 50% commission. GARP outputs are rasterized coverages of the study area. Two different pixel values were used to show the absence or presence of related species; 0 means absence (including areas in which no rules can be applied) and 1 means presence. Then, we calculated each pixel value by summing the "best subset models", using the Raster Calculator function in the Spatial Analyst extension of ArcGIS 9.3, producing final predictions of potential distributions with 11 thresholds (integers from 0 to 10). A pixel was predicted to be present if the threshold ≥ 5 of the 10 best subset models.

We developed a series of tests of model predictive ability. In each case, the developed models and predictions tested were based on independent suites of HFRS data. The HFRS data were divided into quadrants, above and below the median longitude and median latitude: (i) west versus east of the median longitude (hereafter “WE” tests); (ii) north versus south of the median latitude (hereafter “NS” tests); (iii) on-diagonal (upper left-hand and lower right-hand quadrants) and off-diagonal (upper right-hand and lower left-hand quadrants, hereafter “DIAG” tests). In each case, we developed both reciprocal predictions, testing the ability of ENMs to predict the distribution of HFRS in regions where no sampling was available. Finally, data from 2005 through 2008 were used to develop the ENMs, and 2009 data were used to validate the prediction (hereafter “Time” tests).

To investigate relative contributions of the 25 environmental variables, a jackknife procedure was used, which was performed by fitting the model with *n-1* variables and successively omitting one variable. Then, we compared the predictive accuracy of the subset models with the comprehensive model (based on *n* variables), to eliminate variables that lead to over-fitting and to select key risk factors. Occurrence data from 2005 through 2008 were used to perform the jackknife procedure, and the average area under the receiver operating characteristic (ROC)[15] curve (AUC) was used to evaluate predictive accuracy of the model. After the jackknife procedure, the final HFRS prediction was executed again by GARP, using available variables.

### Rodent species composition and HFRS occurrence

**Figure 3:**
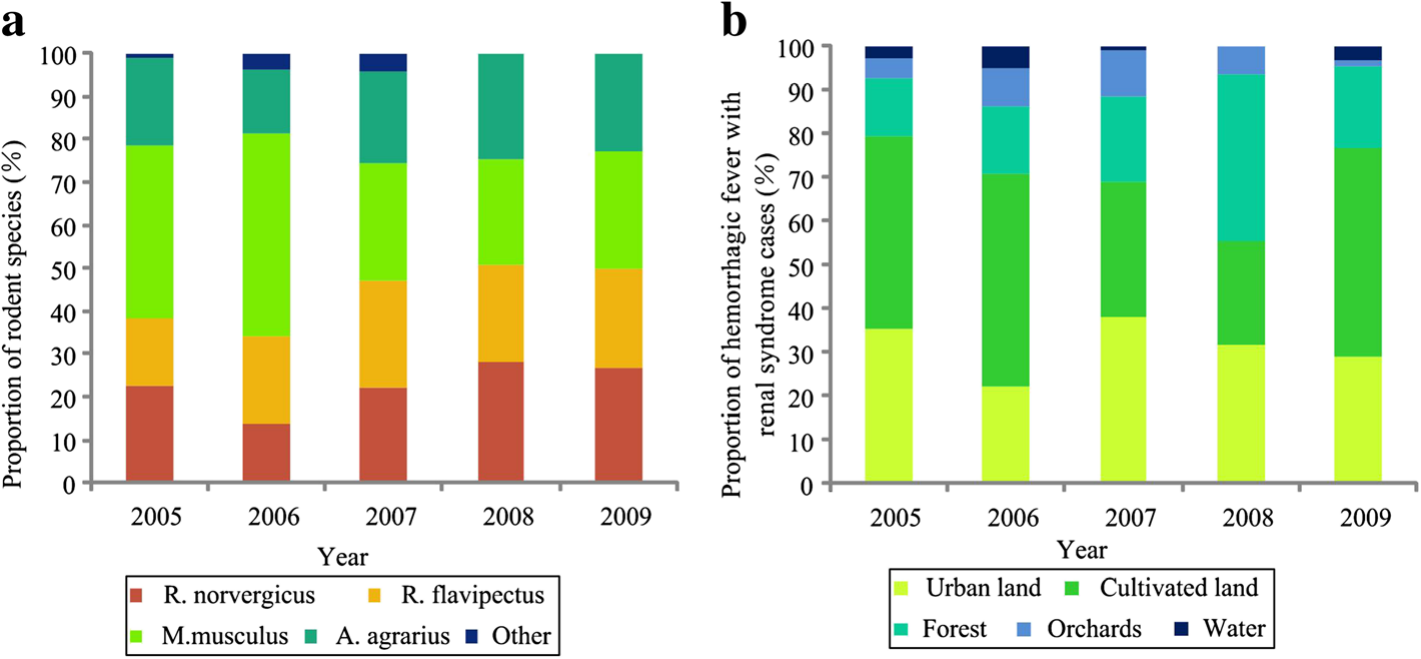
**Composition of rodent species and HFRS cases in Changsha. (a)** Proportion of each rodent species, from monitoring data; **(b)** proportion of HFRS cases in different risk areas.

The relative population density of rodents has important effects on HFRS occurrence. Rodent species composition varies with land use type. Since a certain land cover type is often associated with the presence of hantavirus-infected rodents, hantaviruses are usually associated with distinct rodent species. The annual proportion of HFRS cases was based on five main land use types (cultivated land, orchard, forest, urban and water). We calculated the annual density of various types of rodents (*A. agrarius*, *R. norvegicus*, *M. musculus*, *R. flavipectus* and other species) and annual total rodent density from 2005 through 2009 (Figure 3). Then, we used a Markov chain Monte Carlo (MCMC) method [16] to investigate the relationship between rodent density and HFRS incidence. Matrix R represents HFRS cases in different land use types; rows correspond to years, and columns to HFRS risks. Matrix C represents the density of different types of rodents; rows correspond to years, and columns to rodent density. Then, we calculated the coefficient matrix (Formula 1). Next, the relationship between annual HFRS cases and total rodent density was analyzed using Formula 2. Related environmental variables and rodent monitoring data were used to analyze potential associations between rodent species composition and HFRS incidence in different land use types. The MCMC method was carried out using Matlab software (vR2009b) (MathWorks Inc., Natick, MA, USA)[16].

(1) $$\left(\begin{array}{cccc} R_{11} & R_{21} & \ldots & R_{i1}\\ R_{12} & R_{22} & & \\ \vdots & & \ddots & \vdots \\ R_{1j} & & \ldots & R_{\mathrm{ij}} \end{array}\right)=\beta \cdot \left(\begin{array}{cccc} C_{11} & C_{21} & \cdots & C_{i1}\\ C_{12} & C_{22} & & \\ \vdots & & \ddots & \vdots \\ C_{1u} & & \cdots & C_{\mathrm{iu}} \end{array}\right)$$

(2) $$\psi_{k}=\theta_{1}\cdot \gamma_{k}+\theta_{2},$$

where R_*ij*_ is the proportion of HFRS incidence in risk area *j* in year *i*; *β* is the coefficient matrix; C_*iu*_ is the proportion of relative population density of rodents *u* in year *i*; *γ*_*k*_ is the density of rodents in year k; *ψ*_*k*_ is the number of predicted HFRS cases.

## Results

### Modeling and testing approaches

**Figure 4:**
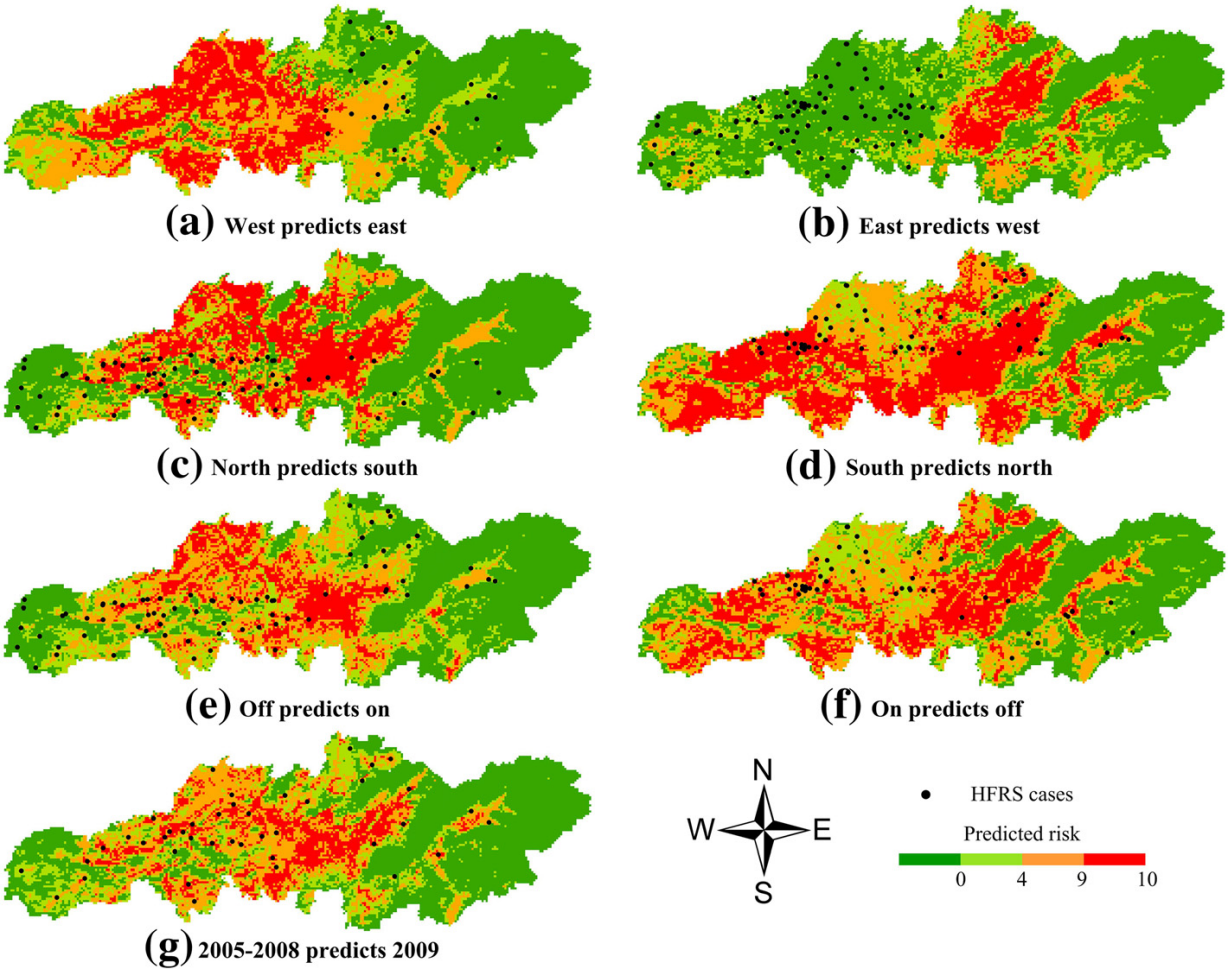
**Examples of spatially stratified tests of ENMs predictions of HFRS distributions in Changsha. (a)** Occurrences in western quadrants were used to predict distributions of cases in eastern quadrants; **(b)** occurrences in eastern quadrants were used to predict distributions of cases in western quadrants; **(c)** occurrences in northern quadrants were used to predict distributions of cases in southern quadrants; **(d)** occurrences in southern quadrants were used to predict distributions of cases in northern quadrants; **(e)** occurrences in off-diagonal quadrants were used to predict distributions of cases in on-diagonal quadrants; **(f)** occurrences in on-diagonal quadrants were used to predict distributions of cases in off-diagonal quadrants; and **(g)** occurrences from 2005 through 2008 were used to predict distributions of cases in 2009.

All tests in this study found that independent sets of test points coincided with ENMs predictions, which were significantly better than random expectations (Table 3). Results of model testing showed that test points were well predicted (at threshold levels of 5–10) in all subsets, except those of the WE testing. In these tests (especially "east predicts west"), fewer test points were correctly predicted, with fewer areas predicted present (Table 3). However, results of all spatial calibration tests (Figure 4) correctly predicted the independent test points, as evident from the significant level of binomial probability (*P*< 0.01). Prediction of the overall study area at one time scale also implied a good model fit (Figure 4 and Table 3).

**Table 3:** Summary of model predictions and tests for Changsha

**Model testing**	**Sample size**		**No. of successes**	**Proportion of predicted area**	**Cumulative bino-mial probability**
	**Train**	**Test**			
NS					
North predicts south	162	165	131	0.3544	<0.01
South predicts north	165	162	151	0.5303	<0.01
WE					
West predicts east	246	81	42	0.1806	<0.01
East predicts west	81	246	6	0.0582	<0.01
DIAG					
On predicts off	157	170	121	0.3001	<0.01
Off predicts on	170	157	137	0.4576	<0.01
Time					
05-08 predicts 09	262	65	62	0.3792	<0.01

Results of the jackknife procedure showed that all environmental variables, except CTI and human population density, contributed to model predictive ability. Exclusion of temperature seasonality, land use, elevation and NDVI (mainly in November, January and June) resulted in the greatest deviations (Table 4).

**Table 4:** Summary of statistical analysis of jackknife procedure

**Variables excluded**	**AUC**^**#**^	**Std Dev**	**Variables excluded**	**AUC**^**#**^	**Std Dev**
Aspect	0.827	.017	NDVI(Jan)	0.806	.018
CTI^*^	0.860	.016	NDVI(Feb)	0.822	.021
elevation	0.817	.018	NDVI(Mar)	0.830	.021
Ecology	0.821	.016	NDVI(Apr)	0.827	.016
Land use	0.813	.016	NDVI(May)	0.839	.017
MHT	0.841	.017	NDVI(Jun)	0.814	.017
Population density^*^	0.855	.016	NDVI(Jul)	0.830	.020
Precipitation	0.821	.017	NDVI(Aug)	0.838	.019
Precipitation seasonality	0.831	.017	NDVI(Sep)	0.824	.017
Human footprint index	0.823	.017	NDVI(Oct)	0.819	.019
Slope	0.833	.018	NDVI(Nov)	0.788	.018
Temperature	0.831	.017	NDVI(Dec)	0.832	.017
Temperature seasonality	0.810	.017			

^**#**^ AUC value of ENMs when variables were excluded.

^*^ Indicates exclusion of this variable from the model to improve its accuracy.

**Figure 5:**
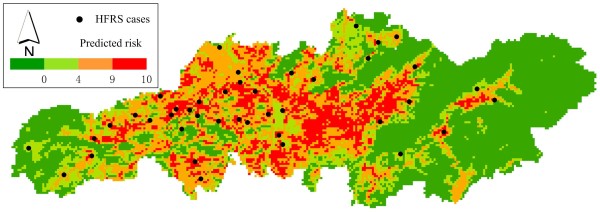
Predicted risk map for HFRS in 2009, overlaid with HFRS case localities in 2009.

Data from 2005 through 2008 were then used to develop the final prediction model, and 65 HFRS cases in 2009 were used to validate the prediction. Population density and CTI were excluded, and 10 best subset models were calculated for a prediction map (Figure 5). The results show that ENMs independently predicted 58 of 65 cases in high risk areas (45 cases were predicted by all 10 subset models) in 2009. Evaluation data were merged with 10,000 randomly selected background points and entered into a ROC analysis, to derive the AUC. The average AUC was 0.855 (95% CI: 0.821–0.886, SD = 0.017, *P*< 0.01), indicating that model performance was satisfactory.

### Ecological characteristics of predicted results

**Figure 6:**
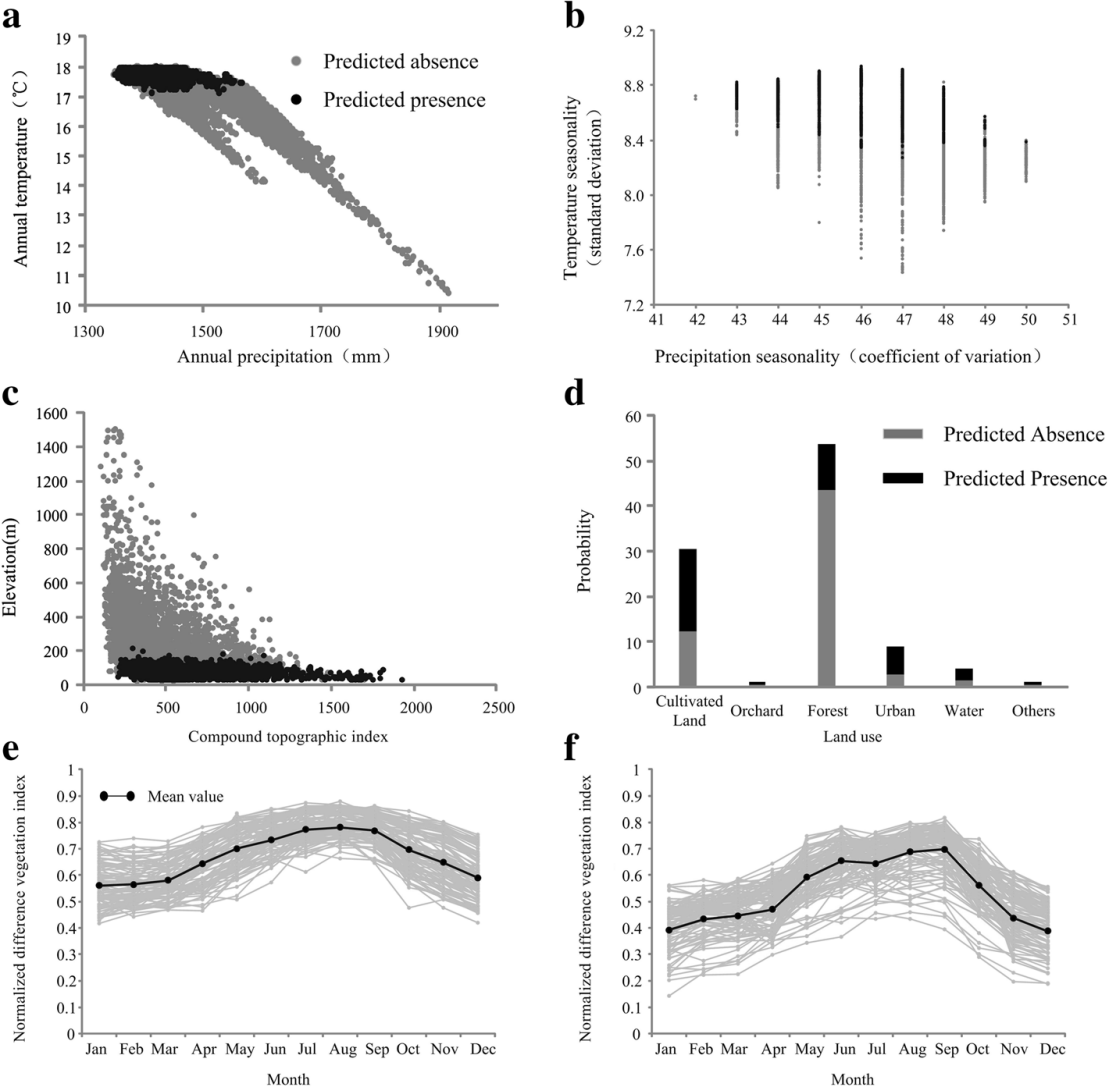
**Exploratory visualization of HFRS niche in two-dimensional environmental space.** Gray indicates available environmental conditions, and black the modeled ecological niche of hantavirus: (**a**) annual precipitation and annual temperature; (**b**) precipitation seasonality and temperature seasonality; (**c**) compound topographic index and elevation; (**d**) land use. Solid line depicts variation in mean monthly NDVI values of (**e**) predicted absent, and (**f**) predicted present.

**Figure 7:**
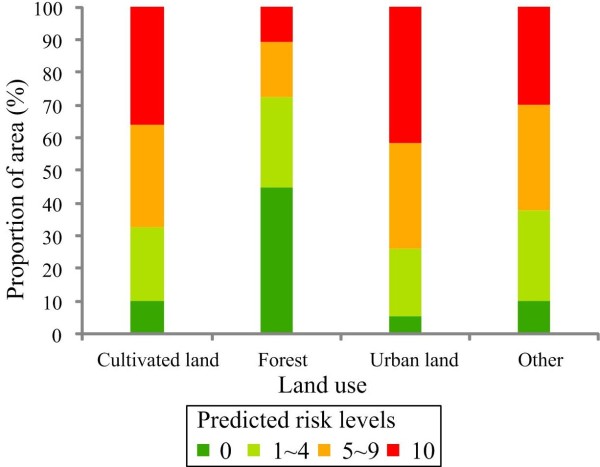
Influence of land use type on HFRS occurrence.

The exploratory visualizations describe ecological characteristics of the predicted results of HFRS occurrence in Changsha, with the 10,000 randomly selected points in areas predicted absent versus present (Figure 6). The results show that HFRS occurrence was restricted to areas of strong temperature seasonality (Figure 6b), with mean annual temperature around 17.5°C (Figure 6a), precipitation below 1600 mm (Figure 6a), and elevation below 200 m (Figure 6c). Risk was concentrated in cultivated and urban land (Figure 6d). The risk level of HFRS is correlated with an increase in area of cultivated and urban land, and a decrease in forested areas (Figure 7). The NDVI value in areas predicted present is lower than in areas predicted absent, but with substantial seasonal variation (Figure 6e and Figure 6f).

### Rodent host and HFRS occurrence

**Figure 8:**
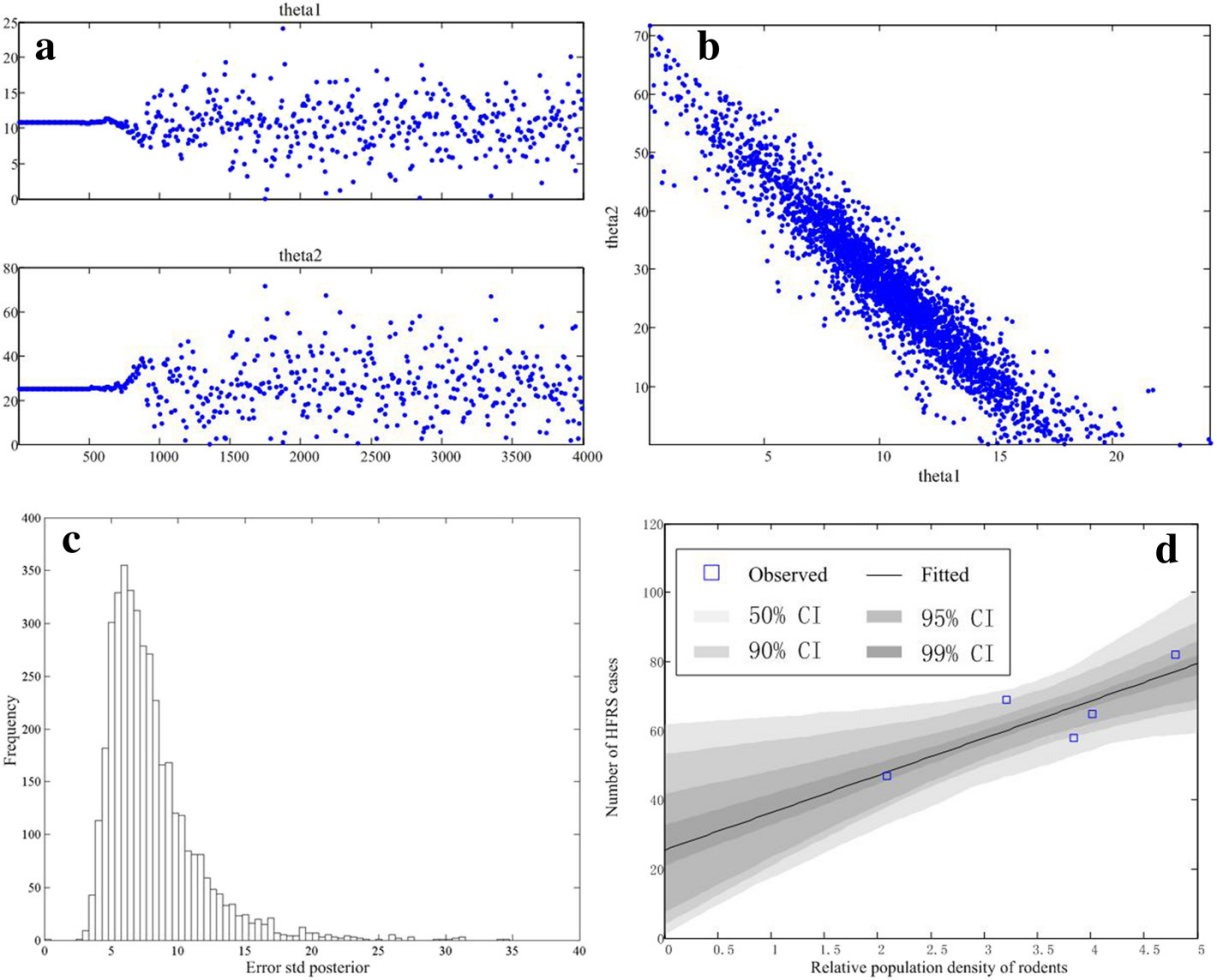
**Association between HFRS cases and relative population density of rodents predicted by MCMC. (a)** Kernel density estimates of posterior distributions; **(b)** pairwise scatterplots of columns of the chain; **(c)** histogram of error standard deviation; **(d)** predictive envelopes of the model.

Correlation analysis showed that the number of HFRS cases was remarkably associated with rodent density (r = 0.84, *P* = 0.07). The prediction model provided reasonably good fits of the relationship between rodent density and number of HFRS cases (Figure 8), in which *θ*_1_ is 10.81 and *θ*_2_ is 25.39. The coefficient matrix used to show the potential influence of rodent species composition on different areas was calculated as

$$\beta =\left(\begin{array}{ccccc} 4.33 & -1.41 & 0.09 & -2.48 & 6.39\\ 29.76 & 0.12 & 2.09 & -35.58 & 16.38\\ -29.39 & 1.99 & -1.18 & 34.81 & -20.70\\ -6.68 & 0.18 & -0.26 & 8.05 & -2.54\\ 2.97 & 0.11 & 0.26 & -3.80 & 1.46 \end{array}\right)$$

The coefficient matrix implies that HFRS cases in urban areas and cultivated land are mainly caused by *R*. *norvegicus* and other species (including *Rattus niviventer*, *R*. *flavipectus*, *Microtus fortis* and shrew species, with total proportion less than 5%), and that risk in forested areas is from *A*. *agrarius*. In terms of population quantity, *R. norvegicus* and *A. agrarius* are considered the dominant species, with varying impacts on HFRS occurrence in different risk areas of Changsha.

## Discussion

With rapid development of the economy and urbanization, the environment in China has changed greatly. Continuous habitat changes for rodent hosts are believed to influence the spatial distribution of HFRS, extending the main epidemic areas from rural to urban areas. This study improves our understanding of the transmission patterns of HFRS. The correlation between rodent species composition and HFRS occurrence also provides a valuable approach for predicting HFRS risk.

Human activities including rapid urbanization, deforestation, agricultural invasion, land use change, pollution and population migration are considered important factors for the outbreak and reemergence of various infectious diseases, and for heterogeneity in the incidence and spatial distribution of these diseases [17-19]. Forest is the most extensive land cover type in Changsha, but the risk level there is low. However, risk levels in cultivated land and urban areas are relatively high.

NDVI is correlated with amount and productivity of vegetation and crops, which are the main food sources for rodents. The NDVI is higher in thick vegetative areas, such as forests and orchards, and lower in cultivated land areas, with significant seasonal variations. HFRS occurrence is closely associated with NDVI. HFRS risk is also affected by differences in local climates. Meteorological factors, including humidity, temperature and precipitation, influence not only the infectivity and vitality of hantavirus but also the distribution of rodent hosts [3]. Moist environments are conducive to the vitality and infectivity of Hantaan virus and to the existence and distribution of rodents. However, too much precipitation destroys the rodent habitat, decreasing the population [4]. HFRS cases are rarely reported in very dry or very wet areas [20]. In Changsha, the high risk areas mostly concentrate in locations with annual temperature around 17.5°C and annual precipitation between 1,300 and 1,600 mm.

The density and species composition of rodent hosts are closely associated with HFRS occurrence. Usually, HFRS incidence escalates with rodent density, and vice versa. Each hantavirus species is predominately associated with a distinct or a few related rodents as its host. Since different rodent species adapt to various environments and habitats, rodent impacts vary by area. This study showed that *A. agrarius* and *R. norvegicus* are the two predominant animal hosts; *A. agrarius* is abundant in forested areas, and *R. norvegicus* is predominant in urban and cultivated areas. Correlation analysis between HFRS incidence and rodent species composition indicates that the main HFRS risks are concentrated in urban and cultivated land and forests, which is consistent with ENMs prediction.

ENMs were used to identify the relationship between HFRS cases and the environment. This was done to explore non-random association between environmental characteristics and known epidemic areas and the entire study area, and to predict potential risks based on the basic ecological demands of HFRS. The MCMC method was used to predict HFRS cases in various areas based on surveillance data of rodent hosts, and cases in diverse land use types. The MCMC results further validated the predictive results of the ENMs. Therefore, this study offers a valuable approach to predict HFRS risk and to analyze transmission patterns in cases when “absence” data are unavailable. The results have important implications for the prevention and control of HFRS. The final prediction model can be used as a guide map for possible future outbreaks in Changsha, and related risk factors may be used as predictors of HFRS occurrence. For the large central area of the map (Changsha County), a predicted high risk area, special measures may needed to prevent the spread of HFRS. Since *A. agrarius* is abundant in forests, and *R. norvegicus* is predominant in urban and cultivated areas, different preventive measures are needed in different areas. All this information provides guidance for public health action.

## Conclusions

The results of temporal testing based on the entire region are clearly better than other spatial calibration tests, and only a few test points were correctly predicted in the WE tests (especially the "east predicts west" test). More applications may be required to investigate ecological differences between HFRS occurrence in the eastern and western areas of Changsha. For optimal performance of temporal testing, time-specific ENMs are suggested for obtaining more specific and detailed disease occurrence information. Limitations of this study should also be acknowledged. The data are from a passive surveillance system, so their quality is not as high as those collected from active surveillance. The environmental variables used in the models are nearly abiotic factors, but in fact, biotic factors are important in disease occurrence. Previous studies have indicated that varying modeling approaches can yield substantially different predictions [21,22]; therefore, selection of modeling methods and environmental variables should be taken into careful consideration. Future work is required to address these issues.

## Competing interests

All authors declare that they have no competing interests.

## Authors’ contributions

HX, XL, JQ, LG, HT were involved in the conceptualization, research design, execution and write-up of the first draft of the manuscript. NL, CH and JZ contributed to database design and data analysis. BC and PZ advised on the study design and the analysis and interpretation of results. All authors were involved in preparation of the manuscript.All authors read and approved the final manuscript.

## Pre-publication history

The pre-publication history for this paper can be accessed here:

http://www.biomedcentral.com/1471-2334/13/305/prepub
